# Improvement of hand functions of spinal cord injury patients with electromyography-driven hand exoskeleton: A feasibility study

**DOI:** 10.1017/wtc.2020.9

**Published:** 2021-01-05

**Authors:** Youngmok Yun, Youngjin Na, Paria Esmatloo, Sarah Dancausse, Alfredo Serrato, Curtis A. Merring, Priyanshu Agarwal, Ashish D. Deshpande

**Affiliations:** 1 Department of Mechanical Engineering, The University of Texas at Austin, Austin, Texas, USA; 2 Department of Mechanical Systems Engineering, Sookmyung Women’s University, Seoul, Republic of Korea

**Keywords:** electromyography, grasp, hand exoskeleton, spinal cord injury

## Abstract

We have developed a one-of-a-kind hand exoskeleton, called Maestro, which can power finger movements of those surviving severe disabilities to complete daily tasks using compliant joints. In this paper, we present results from an electromyography (EMG) control strategy conducted with spinal cord injury (SCI) patients (C5, C6, and C7) in which the subjects completed daily tasks controlling Maestro with EMG signals from their forearm muscles. With its compliant actuation and its degrees of freedom that match the natural finger movements, Maestro is capable of helping the subjects grasp and manipulate a variety of daily objects (more than 15 from a standardized set). To generate control commands for Maestro, an artificial neural network algorithm was implemented along with a probabilistic control approach to classify and deliver four hand poses robustly with three EMG signals measured from the forearm and palm. Increase in the scores of a standardized test, called the Sollerman hand function test, and enhancement in different aspects of grasping such as strength shows feasibility that Maestro can be capable of improving the hand function of SCI subjects.

## Introduction

The number of spinal-cord-injury (SCI) patients in the United States was estimated to be 282,000 in 2016 (National Spinal Cord Injury Statistical Center, 2005). Approximately 45% of SCI patients have residual function in their arms and shoulders, but patients who have partially or totally lost their hand-control ability are unable to perform activities of daily living (ADL). Reduced grasping power and the inability to control the movements of the hand result in frustration because patients are unable to perform tasks such as grasping, lifting, and manipulating an object. Assistive exoskeletons have the potential to improve the quality of the life of the SCI patients by powering their hand movements to fulfill daily tasks. Active hand exoskeletons have been developed to provide assistance for hand function (Heo et al., 2012; In et al., 2015; Zhou et al., 2019; Ferguson et al., 2020; Tran et al., 2020).

To provide assistance for functional tasks with hand, the control and communication between the exoskeleton and the user must allow for seamless and reliable transfer of information so that the motion intent is received and fed to the robot controller to achieve the desired action. The surface electromyography (sEMG) signal has been used to extract motion intents in exoskeleton studies. Several researchers have developed sEMG-driven hand exoskeletons (Benjuya and Kenney, 1990; Dicicco et al., 2004; Zhao et al., 2016; Lu et al., 2017; Thielbar et al., 2017; Lu et al., 2019). This signal can be noninvasively measured on the target muscles relevant to desired tasks and the operation of the assistive device is not disturbed by the motions of other body parts (Lenzi et al., 2012; Tigra et al., 2018). However, these studies have mostly operated the exoskeletons in simplified motions such as 1-degree of freedom (DoF) actuation with a binary threshold for opening and closing the fingers.

There are major challenges in design and control for developing an active assistive exoskeleton for hand function. The design of an exoskeleton needs to be light and allow for comfortable use for long periods of usage. Moreover, the actuation of exoskeleton needs to be compliant to prevent harming the subject’s hand due to rigid control. The second major challenge is in exoskeleton control to recognize the intention of the subject and deliver the appropriate hand pose safely to complete a task. If an active device fails to reliably identify the intention, it may actively hinder movements of subjects. Under controlled conditions, many hand poses could be successfully estimated using different algorithms approaches with sEMG signals (Micera et al., 2010; Simao et al., 2019). However, the relation between electromyography (EMG) signals and hand poses is very complex in real-world scenarios. For example, the orientation of the forearm, the shape and weight of the object being handled, fatigue and emotional level of the subject can all show important effects in EMG classification during grasping tasks in real-time EMG-driven control (Kim et al., 2018). In addition, for SCI patients, the use of sEMG is more limited than healthy persons because the degree of paralysis varies depending on the individual level of injury and the sEMG signals are noisy and weak (Dietz et al., 2009).

To achieve assistance through an EMG-driven exoskeleton in daily living, one of our underlying ideas is that an EMG-driven exoskeleton for SCI patients can provide effective assistance in hand function only using a few hand poses given the exoskeleton has compliance at its joints and uses a robust classification approach. This idea may abate the challenge of classification of EMG signals, as the algorithm would not need to classify EMG signals into many different grasping modes. Maestro, developed to provide assistance in hand function for SCI patients (Agarwal et al., 2015; Agarwal and Deshpande, 2017; Agarwal et al., 2017), was used for the preliminary experiments to provide assitive poses. In our previous studies, limiting the number of target grasping modes to a few hand poses estimated with a small number of electrodes were shown to be more advantageous for assistive purposes by simplifying the set up and control (Yun et al., 2017). In addition, to enhance robust successful control of the exoskeleton, a majority vote classifier was used to generate control inputs from the classified motion intents (Yun et al., 2017). Although our proposed methodologies showed the feasibility to provide assistance in pilot experiments for SCI patients, thorough quantitative analysis was not given featuring a comparison between online and offline performance of the classifier and the control approach during actvities of daily living.

In this study, we quantitatively evaluated the performance of the classifier and the proposed control approach for detecting the grasp intention in real-time in healthy subjects and SCI patients. We also utilized a standardized hand function test, called Sollerman hand function test (SHFT) to evaluate the performance of the proposed method of EMG-based robotic exoskeleton in assisting the hand function for the SCI patients. This study is the first of its kind that features a full system that takes biological signals from the SCI patients and assists them seamlessly in achieving daily grasping and manipulation tasks in real-time. To assess the functionality of our proposed control approach, we compared both offline and online classification results in producing input commands to the robot for a sample of healhty subejcts. The results of SHFT demonstrate that the SCI subjects were able to operate the assistive device by themselves despite their limited muscle activations. Moreover, the hand function of C6- and C7-level SCI subjects were improved when the subjects performed the test with the proposed method using Maestro.

## Methods

We utilized Maestro exoskeleton (see Section “Assistive hand exoskeleton: Maestro”) to provide assistance for grasping and manipulation tasks. A minimal set of hand poses were selected from the eight most frequently used grips in ADL considering the compliance of the robot (see Section “Target hand poses for Maestro”). The locations of sEMG sensors were optimized for SCI patients (see Section “Locations of sEMG sensors”). EMG-based classification was used as an assistive controller for Maestro (see Section “Classification”). The classification results were used to control Maestro based on a probabilistic approach for stable operation (see Section “EMG-based Maestro control”).

### Assistive hand exoskeleton: Maestro

**Figure 1.**
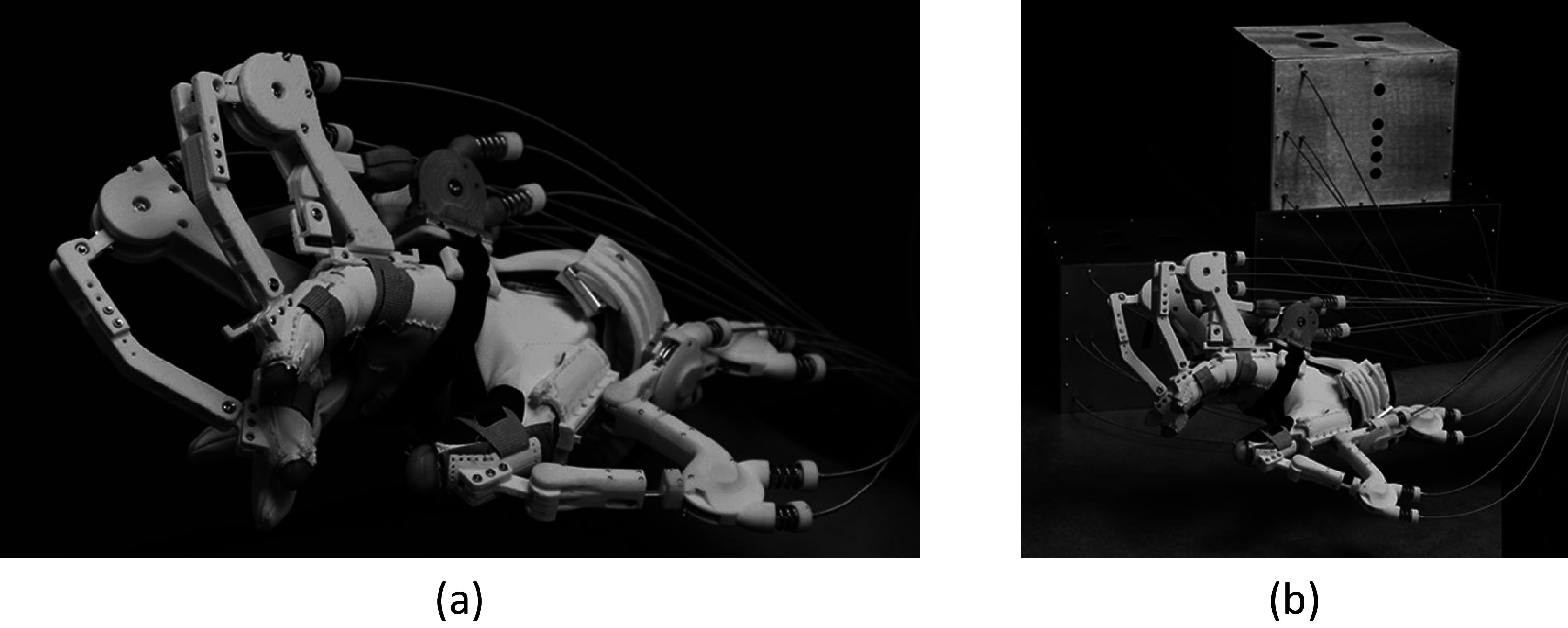
Developed hand exoskeleton (a) Maestro and (b) overall system for Maestro.

Maestro was utilized to provide assistance to the hand as shown in Figure 1. Maestro satisfies many requirements for an active assistive hand orthosis, including light weight, comfort in wearing, compliance in actuation, and the capability to generate essential hand poses.

Maestro has eight DoFs: four active DoFs for the thumb and two active DoFs for the index and middle fingers, which facilitate various hand poses required in ADL. For the thumb, abduction/adduction of the carpometacarpal (CMC) joint and flexion/extension of the CMC, metacarpophalangeal (MCP), and interphalangeal (IP) joints were actuated. For the two fingers, flexion/extension of the MCP and proximal interphalangeal (PIP) joints were actuated. According to hand ergonomics studies (Sollerman, 1980; Tavakoli et al., 2015), a significant portion of the tasks in ADL are performed by the thumb, index, and middle fingers. For example, a housemaid and a machinist were able to perform approximately 80 and 70% of grasping tasks, respectively, utilizing only these three digits.

Exoskeleton joints were actuated by a pull-pull mechanism utilizing Bowden cables. Electric motors were remotely located and the pulleys on the motor shafts were connected to the actuated exoskeleton joints. Because the motors are located remotely, the weight of Maestro is significantly reduced while maintaining sufficient power capacity for performing grasping actions. The actuation of Maestro on the hand is compliant because the remote electric motors are position-controlled by series-elastic-components and compression springs. Therefore, a subject is able to interact with various objects, even if the target hand pose of Maestro is somewhat different than the required hand pose for a task. Detailed actuator information (e.g., optimal stiffness of compression springs and characterization of Bowden cable friction) is presented in Chen et al. (2014).

### Target hand poses for Maestro

In a study by Sollerman (1980), eight most frequently used grips are identified for ADL in healthy humans: transverse volar grip, spherical volar grip, lateral pinch, diagonal volar grip, extension grip, tripod grip, five finger pinch, and pulp pinch. They have also developed a standardized hand function test to measure the overall function of the hand in ADL. The test consists of 20 subtests and each subtest is scored by the examiner from four to zero points according to the guidelines as shown in Table 1.

**Table 1.** SHFT scoring guide

Criteria	Score
The task was completed with no difficulty within 20 s and with the prescribed hand grip of normal quality.	4
The task was completed, but with slight difficulty, or the task was not completed within 20 s, but within 40 s, or the task was completed with the prescribed hand grip with slight divergence from normal quality.	3
The task was completed, but with great difficulty, or the task was not completed within 40 s, but within 60 s, or the task was not performed with the prescribed hand grip	2
The task was only partially performed within 60 s.	1
The task could not be performed at all.	0

However, it is neither desirable nor practical to use all eight hand grips as the set of commands for Maestro in assisting a SCI subject. The main reason for this is that several hand grips share similar muscle activity patterns and sEMG sensors cannot discriminate between individual muscle signals, especially in subjects with existing neuromuscular conditions (Na et al., 2017). Previous research (Liu and Zhou, 2013) has shown that some hand poses can be reliably classified based on sEMG signals, while others may be confused with each other. This incorrect classification is particularly undesirable when controlling an active device because it results in oscillation between the misclassified hand poses. Furthermore, it could result in unstable grasps and drops during object manipulation. Additionally, the shapes of the objects and stiffness of the hand joints also affect the choice of hand poses (Friedman and Flash, 2007). Thus, a similar set of kinematic control commands for the hand may result in two different types of interactions depending on the geometrical and material properties of the hand and the object being manipulated.

**Figure 2.**
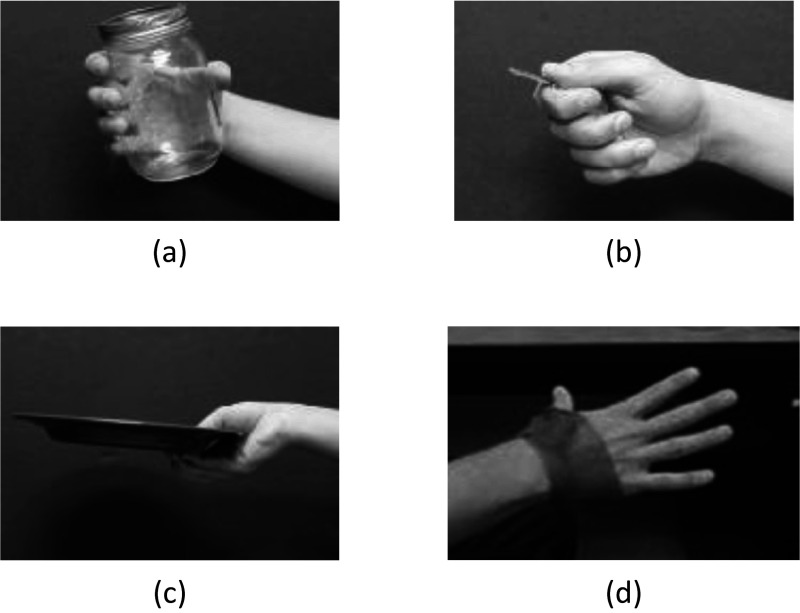
Four selected poses: (a) transverse volar grip, (b) lateral pinch, and (c) extension grip chosen from the eight most frequently used grips in ADL (Sollerman, 1980), as well as the (d) extension pose for Maestro control.

In our study, we have selected four target hand poses taking into account the compliance properties of the exoskeleton as shown in Figure 2, while preserving the ability to grasp all the daily objects listed in the test. To implement the target poses continuously and robustly, each grasping pose was required to be performed proceeding the extension pose. For example, if a subject wanted to make a lateral pinch when the current pose was a transverse volar grip, the extension pose was first performed, followed by the lateral pinch.

**Figure 3.**
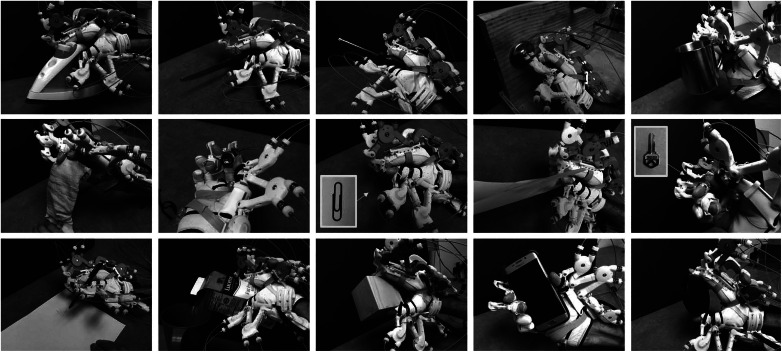
A C5/C7 incomplete SCI subject who could barely generate flexion of the index and middle fingers was able to grasp the 15 objects listed in the SHFT. In this experiment, only the four target hand poses of Maestro were utilized.

Finally, we performed the standardized SHFT to evaluate the grasping performance of SCI patients. In this study, the target hand poses for Maestro were the transverse volar grip, lateral pinch, and extension grip, which are three of the eight most frequently used grips in ADL (Sollerman, 1980). Our design is validated by our preliminary study (Yun et al., 2017), in which we confirmed that a C5/C7 incomplete SCI subject with a compliant hand orthosis was able to grasp 15 objects used in ADL utilizing only a minimal set of hand poses, including the transverse volar grip, lateral pinch, and extension grip (Figure 3). The basic premise of the experiment was that a researcher increased the number of the target hand poses or replaced a target hand pose with another until the SCI subject was able to grasp all 15 objects (see details in Yun et al., 2017). This significant reduction in the number of target poses for ADL is advantageous for sEMG signal classification because a smaller number of classes generally leads to a higher success rate in classification problems.

### Locations of sEMG sensors

For intention recognition, we preferred to select task-relevant muscles to make the voluntary robot operation more intuitive for the user. The precise sensor locations were identified by palpating the right forearms and palms of the subjects while asking them to make an effort to contract their muscles or move their fingers. For more severe SCI cases, depending on their injury, the locations of the sEMG sensors may need to be adjusted to target muscles that are innervated at higher spinal levels or muscles that are more available, such as the wrist extensors or flexors, based on the level of injury. The final decisions regarding sensor placement were made based on the anatomy of the muscles for hand functions and discussions with an occupational therapist with an SCI specialty. EMG sensors must be able to measure the muscle activity of the muscle groups that are responsible for generating the target hand poses and are available for individual SCI subjects.

**Figure 4.**
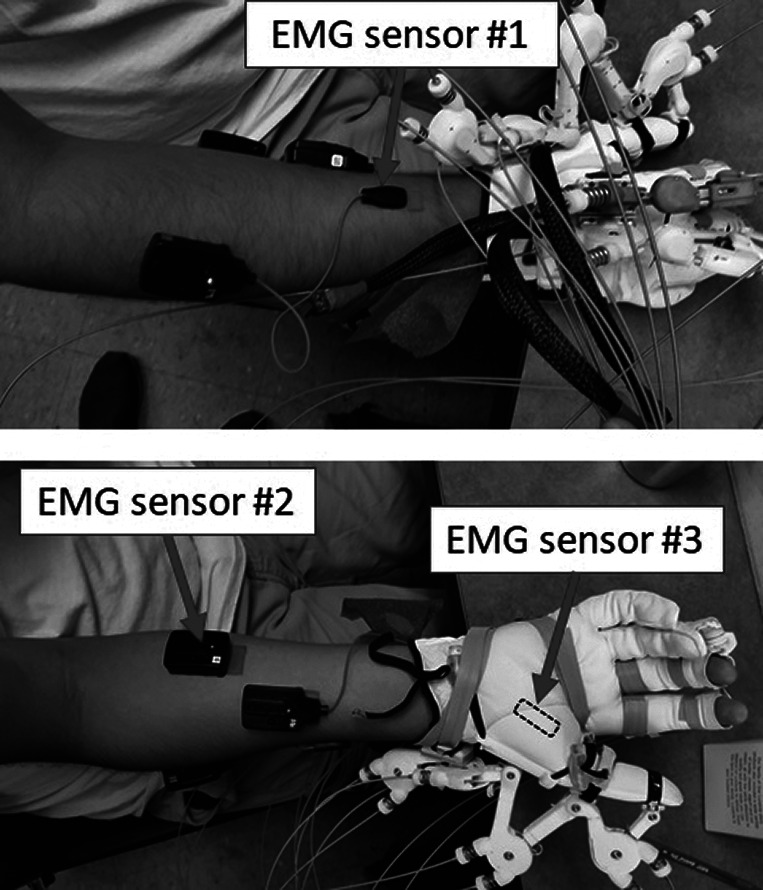
Three wireless sEMG sensors were utilized to identify the intentions of the SCI patients. Three sensors detected the flexion of the fingers, extension of the fingers, and thumb flexion and abduction.

We selected three sEMG sensor locations for the classification of motion intentions following the above considerations. The three sEMG sensors targeted the muscles flexor digitorum superficialis (FDS) and extensor digitorum (ED) on the right forearm and the combined signal from the flexor pollicis brevis (FPB) and abductor pollicis brevis (APB) (the FPB and APB are very close to each other) on the palm (Figure 4). Based on the results from our preliminary testing (Yun et al., 2017), the utilization of more than three sensors showed no significant improvement in classification performance for the four target poses with relaxation. The optimal number and locations of sEMG sensors were determined through the same experimental protocol for two healthy subjects and one SCI patient. Therefore, in this experiment, three sEMG sensors were utilized to classify three target grasping poses, extension, and relaxation.

### Classification

The sEMG signals were measured with 2 kHz sampling rate and were amplified 1,000 times from three electrodes on the forearm and the hand as shown in Figure 4. The signals were processed to extract the linear envelope for classification using the following procedure. The offsets of the signals were removed compared to the relaxed state and the signals were rectified to obtain magnitude values. Next, a third-order Butterworth low-pass filter (cutoff frequency of 4 Hz) was applied to produce the linear envelope representation of the signals (Dicicco et al., 2004; Kwon et al., 2014). This low-pass filter resulted in an approximately 100 ms delay. Finally, the signals were normalized based on the maximum voluntary isometric contractions (MVICs) of the relevant muscles. The amplitudes of the three rectified and filtered EMG signals at each time step are used to train an artificial neural network (ANN) algorithm to classify five classes of hand poses.

**Figure 5.**
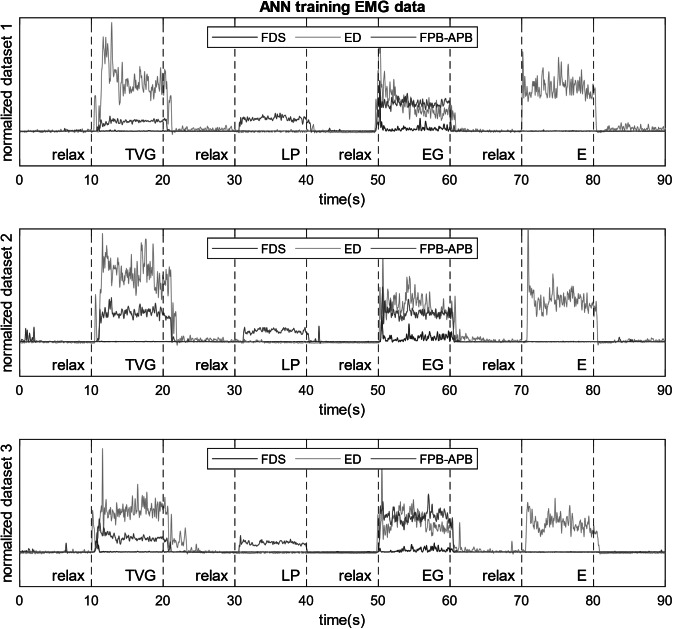
Postprocessed signals extracted from three sensors during training of ANN for hand pose classification. Targeted muscles signals are from FDS, ED, and a combination of FPB and APB. The differences in signal trends between different hand poses, and the similarity of signals of a specific hand pose between trials make it possible to perform intention recognition through the ANN.

The five classes consist of the four target hand poses and relaxation, as shown in Figure 2. One example of the time series data (rectified, normalized, and filtered) collected during three trials which were used for training of the ANN for a healthy subject is shown in Figure 5 highlighting the separability of the classes using signals from the three sensors. We selected a two-layer feed-forward network with sigmoid hidden layer and softmax output neurons. The network model was trained via scaled-conjugate-gradient backpropagation.

When the patients could not complete a specific pose because of symptoms of SCI, we asked them to perform the pose as best as they can. The data from the incomplete poses were also used for classification same as the other poses. The target poses were performed by Maestro based on the results of classification and the predefined exoskeleton joint angles were generated to match the grasping class detected by the ANN.

### EMG-based Maestro control

The goal of the control system is to generate appropriate commands for Maestro based on sEMG signals of the SCI patients to select the intended target hand poses and fulfill the grasping tasks in ADL. The overall EMG-based Maestro control is executed through the following steps. Customized joint angle values for each grasp are selected upon donning of the exoskeleton to each subject’s hand. The EMG signals are recorded and processed (offset removal, rectification, low pass filter) using a LabView program on a separate computer and the filtered EMG signals are communicated to the hand exoskeleton through User Datagram Protocol (UDP). An ANN program is trained and created for each subject based on their training data and is then transferred to the Maestro computer for real-time robot control. The EMG signals are received on the Maestro computer (National Instruments CompactRIO), where the classification is performed using the trained ANN and the hand pose command is selected as detailed in the following paragraphs. Finally, the appropriate position commands are sent to motors actuating each joint of the exoskeleton that correspond to the selected hand pose.

If not handled properly, the noise in EMG signals and movement of the arms and wrist can cause problems during classification and may lead to frequent fluctuations between the hand poses. For the operation of an active assistive device, in contrast to gesture recognition, we require a higher success rate for EMG classification to ensure safety requirements in physical interactions. To improve the success rate and address the problems mentioned above, the Maestro controller utilizes a probabilistic approach (Yun et al., 2017). This approach utilizes three main strategies to enable safe and robust robot control. Firstly, the Maestro controller records the classification results obtained from the ANN for a defined time window of 0.05 s or 100 classification samples, then counts the classification results for that window. Once the count of a classification result exceeds a certain threshold (chosen on a subject-specific basis), the Maestro controller changes the target hand pose. This probabilistic approach filters out incorrect classification results caused by EMG noise or the transition of muscle states.

**Figure 6.**
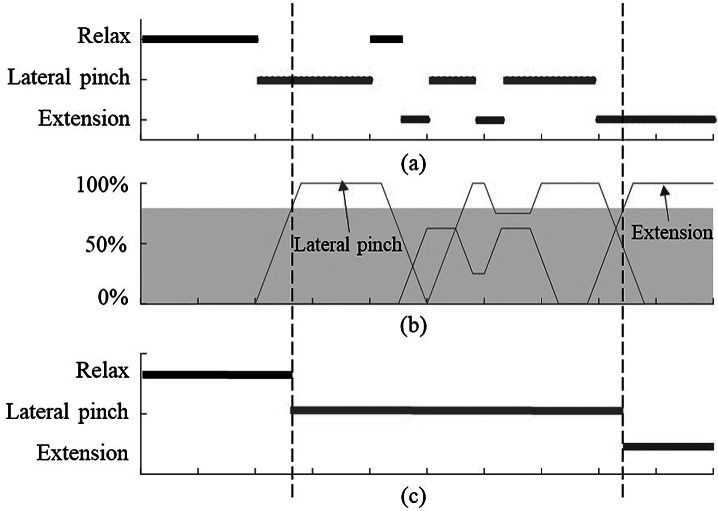
Conceptual control mode changes in Maestro corresponding to EMG classification results; (a) the EMG classification results obtained by the ANN, (b) the relative frequency of classification results, (c) the target hand poses of the Maestro controller.

Figure 6 presents the conceptual control model for only flexion and extension poses. The change in target hand pose is made when the relative frequency of a classification result exceeds a defined threshold. First, the target hand pose changes to lateral pinch when the frequency of lateral pinch is greater than the threshold. If the subject relaxes their muscles and transitions to extension, the classification results are noisy, but the target hand pose is maintained as lateral pinch. Finally, when the frequency of extension exceeds the threshold, the target hand pose is changed to extension. Based on this probabilistic approach, Maestro’s decisions are robust to occasional classification errors.

The second strategy, which was implemented mainly because of SCI subjects with weak muscle signals, was enforcing a requirement to pass through extension pose before switching between two grasping poses. We realized during the experiments that the signals for extension pose was distinctly different than the rest of the hand poses. However, there was a higher probability of confusion between other hand poses. In order to prevent sudden involuntary change between grasping poses during object interactions, the Maestro control program requires subjects to switch to extension pose before trying a different grasping pose.

Thirdly, to prevent fatigue and ensure ease of use for SCI patients, the patient could relax their muscles after they have switched to a classified hand pose. After the selection of a hand pose based on the first mentioned strategy, the probabilistic control approach would not switch from a grasping pose or extension pose once the relaxed state was detected.

## Experimental Validation of the EMG-Based Classification and Assistance

We validated the proposed methodologies in a series of experiments with healthy and SCI subjects. The ANN classification approach and the probabilistic control approach were tested and validated on two healthy subjects to ensure the success of the control approach during static and dynamic conditions. The experiment with SCI subjects helped to validate the feasibility and performance of Maestro and the proposed control approach as an active assistive device to help SCI subjects with ADL.

### Subjects

We recruited two healthy subjects (ages 34 and 35) for validating the ANN classification and the pobablistic approach, and four SCI patients to evaluate the sEMG-based assistance performance of Maestro. They provided written informed consent prior to any study-related procedures. Detailed information regarding the subjects is provided in Table 2. The SCI subjects were diagnosed with complete or incomplete (C5–C7) spinal cord injuries as defined by the American Spinal Injury Association impairment scale classification. They did not have any other conditions (e.g., severe arthritis or extreme shoulder pain) that would interfere with valid administration of our measures or with interpreting motor testing results.

**Table 2.** SCI patients who participated in the experiment

Subject no.	Gender	Hand dominance		Age	Age at injury	Injury level
		Pre-injury	Post-injury			
S01	M	R	R	57	53	C5/C7 incomplete
S02	M	R	R	34	29	C6 incomplete
S03	M	R	R	59	52	C6
S04	M	R	R	51	33	C5

All experiments were conducted with approval from the institutional internal review board.

### Experimental procedures

#### Preparation

In this research, we have used the Delsys Trigno™ Wireless EMG system to collect muscle activation data to train the ANN program and operate the hand exoskeleton through intention recognition. Three EMG sensors were attached to the forearms and palms of the subjects. Proper muscle locations were identified by palpating the right forearm and palm. The EMG sensor locations for the experiment are presented in Figure 4. The EMG sensors were securely protected by wrapping the forearm with fabric strips and then covering them with a tubular bandage. Next, the MVICs of the hands of the subjects were measured. Each patient’s hand was placed and secured in a hand splint to measure the MVIC of each muscle. A custom-made splint was utilized to measure the MVICs of the hand muscles of the SCI patients. Each subject was asked to perform maximum finger flexion, finger extension, and thumb flexion while their muscle activity was displayed to them on a computer screen. The MVICs measured in this stage were utilized to normalize the EMG data during the preprocessing of sEMG signals.

#### Training algorithm

The subjects were asked to perform multiple trials (four for healthy and three for SCI) with five different hand poses for interacting with real objects while their muscle activities were recorded. The tasks were holding a jar (transverse volar grip), holding a key (lateral pinch), holding a plate (extension grip) (Figure 3), relaxing the hand, and extending the fingers and thumb. If a subject was unable to complete a task because of their SCI, they were asked to perform the task to the best of their ability. Each task was performed for 10 s and after finishing a task, relaxation of the muscles was performed for 10 s. To preserve the accuracy of the recorded EMG data and eliminate the effects of transitioning between different grasp modes, the 2 s at the beginning and end of each grasp were discarded and only the middle 6 s were utilized to build the classification algorithm. We randomly selected 70% of these data from the three trials to train the neural network algorithm, and we used the remaining 30% for cross-validation. Additionally, for healthy subjects, we randomly used 70% of training data from three sets to train the ANN and we tested the classification performance first on the remaining 30% of data and then on a separate dataset.

#### Testing the probabilistic classification algorithm

Testing the probabilistic approach for real-time nontrained grasping of daily objects was done on two healthy subjects. In this experiment, after the attachment of the sEMG sensors, and training the ANN, the subjects were shown visual cues and asked to perform a sequence of grasping of six objects used in the SHFT that are representative of the daily objects and essential grasps (Figure 3). The subjects began with a relaxed pose, then started grasping the objects one-by-one for 10 s. The objects used in this experiment were a cylinder (cup), screwdriver, coin, key, pen, and envelope. After grasping each object for 10 s, the subjects switched to extension pose for 5 s and then relaxed their muscles for 5 s before initiating the next object grasp. We evaluated the success of the proposed probabilistic approach in detecting and robustly maintaining the intended grasping poses during real-time object interaction.

#### Control practice

The SCI subjects wore the Maestro device and a researcher adjusted the link lengths of the device to fit the individual hand sizes of the subjects and ensure comfort. We then customized the target hand poses of the Maestro controller, namely the transverse volar grip, lateral pinch, extension grip, and extension. We performed this by empirically adjusting the commanded robot joint angles required for each hand pose considering the subject’s hand size and physical condition (such as muscle spasticity or flaccidity) until we achieved the desired hand pose. Note that the corresponding joint angle commands at the exoskeleton result in different output torque values at the exoskeleton and finger joint through the series elastic actuator (SEA) for different object shapes and sizes and various hand conditions of the subjects.

The subjects were asked to familiarize themselves with the system by controlling the Maestro device utilizing their muscle activation. Following the customization of the Maestro settings, the EMG-driven controller was turned on and the subjects had 20 minutes to practice controlling the Maestro device with their muscle activity and interact with a few provided objects. To use the proposed system, the subjects needed to generate the sEMG signals using their muscles. Through the classification and the control approach, the target poses were selected and delivered by the Maestro exoskeleton.

#### Sollerman hand function test

The effectiveness of the proposed methodology was validated based on the hand function of SCI patients evaluated in a standardized hand function test. The hand function performance with and without Maestro was evaluated based on a test called the SHFT (Sollerman and Ejeskär, 1995). The SHFT was developed to evaluate the hand functions of tetraplegic patients in ADL. Compared to other evaluation methods, such as the graded redefined assessment of strength sensibility and prehension (GRASSP) (Kalsi-Ryan et al., 2012) and the Toronto Rehabilitation Institute hand function test (TRI-HFT) (Kapadia et al., 2012), SHFT is more focused on the evaluation of hand functions in daily activities with objects utilized in daily tasks, rather than evaluating the individual components of hand functions, such as muscle strength, sensibility, and motor coordination, which are the main factors of GRASSP and TRI-HFT. We selected the SHFT for our experiments because this evaluation is focused on the hand functions of SCI patients in ADL.

**Figure 7.**
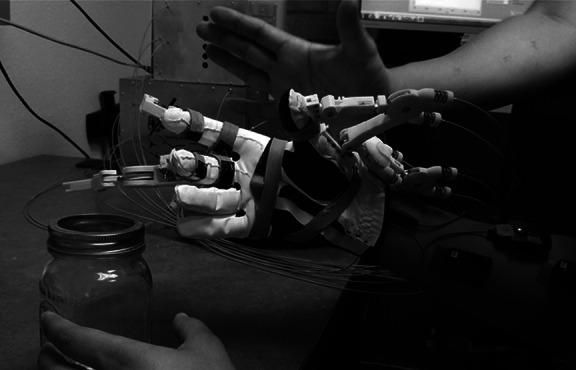
Transverse volar grip performed by an instructor to demonstrate how to grasp an object for an SCI patient with Maestro.

The SHFT evaluates the hand function of subjects based on 20 tasks inspired by ADL. Each subtest is scored on a scale of 0–4 based on various scoring criteria, including time to complete a task, successful completion of a task, utilization of a normal hand grip, and number of drops. The maximum score for the SHFT is 80. The tasks to be performed include closing and opening zippers, picking up coins, operating a screwdriver, writing with a pen, pouring water from a jug, and lifting an iron (Figure 3). A brief description of these tasks is provided in Table 3. During the experiment with SCI subjects, a researcher described the SHFT and its scoring criteria to the subjects as shown in Table 1. The subjects sat at a table with its height adjusted for their wheelchair and the SHFT kit was placed on the table. A researcher performed and demonstrated each task in the SHFT utilizing a normal grasping mode and asked the subject to attempt to perform the same task utilizing the correct grasping mode (Figure 7). In this manner, the subjects completed the SHFT utilizing Maestro. An occupational therapist observed and timed each task and scored the tasks on a scale of 0–4 based on the scoring guide. After each patient completed the SHFT with Maestro, we removed the Maestro device and the EMG sensors, and the subjects rested for 10 minutes. The subjects then attempted the SHFT with their bare hands. Before each task, a researcher again performed and demonstrated the task utilizing a normal grasping mode and asked the subjects to attempt the same task.

**Table 3.** SHFT scores for four SCI patients with Maestro (w/ Exo) and without Maestro (w/o Exo)

Task description	S01 (C5/C7 in.)			S02 (C6 in.)		S03 (C6)		S04 (C5)	
	w/o Exo	w/ Exo 1st	w/ Exo 2nd	w/o Exo	w/ Exo	w/o Exo	w/ Exo	w/o Exo	w/ Exo
Pick up key, put into Yale-lock and turn 90°.	1	1	1	3	2	1	1	1	1
Pick up coins from flat surface and put coins into purses mounted on the wall.	2	2	2	2	3	3	1	1	1
Close and open zippers.	2	1	1	2	2	2	1	1	1
Pick up coins from purses.	1	0	1	2	1	1	1	1	1
Pick up wooden blocks, lift over edge.	3	2	3	2	3	3	3	2	1
Lift iron over edge.	2	4	4	2	4	3	4	3	4
Turn screw with screwdriver.	2	2	3	3	3	2	3	1	1
Pick up nuts and put nuts on bolts.	1	1	1	2	1	1	1	1	1
Unscrew lids of jars.	2	3	2	2	2	2	3	2	2
Do up buttons.	2	1	1	1	1	1	1	1	1
Cut Play-Doh (plasticine).	2	2	1	2	2	2	1	2	1
Put elasticated tubular bandage on the other hand.	2	3	3	2	1	1	1	1	1
Write the word “name” on paper.	2	4	4	4	3	2	3	3	4
Fold paper, put into envelope.	2	1	2	2	1	1	1	3	1
Put paperclip on envelope.	2	3	3	2	3	3	1	1	3
Pick up telephone receiver and bring it to the ear.	3	3	4	3	3	3	2	3	2
Turn door handle 30°.	4	3	4	3	3	3	4	3	4
Pour water from 1 L paper milk or juice package (pure-pak carton).	2	4	2	2	3	2	4	1	1
Pour water from jug.	2	3	4	2	4	2	2	1	1
Pour water from cup.	2	4	4	2	4	2	3	1	1
Total score	41	47	50	45	49	40	41	33	33

S01 performed the tasks twice with Maestro (w/ Exo 1st and w/ Exo 2nd).

## Results

### Classification

In this section, we presented the classification results for the healthy and SCI subjects, as shown by the confusion matrices from the ANN algorithm.

#### Healthy subjects

**Figure 8.**
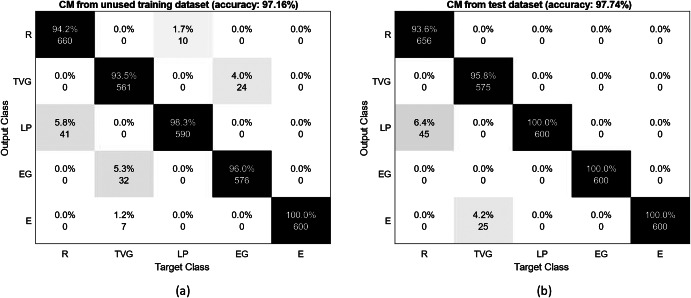
Confusion matrices for the healthy subject (HS01), tested on (a) the remaining data from the initial three datasets and (b) data from a separate dataset. R, TVG, LP, EG, and E are relaxation, transverse volar grip, lateral pinch, extension grip, and extension, respectively.

We evaluated the performance of the sEMG-based classification from the ANN trained by 70% of data randomly selected from three training data sets. Figure 8 demonstrates the results of validating classification performance of the trained ANN on the remaining data from the training datasets for the first healthy subject (Figure 8a) and on a separate dataset collected at a different time from the subject (Figure 8b). In both conditions, the extension pose was most reliably classified with an accuracy of 100%. The overall classification accuracy was very close in both cases, 97.16% for unused data from the training datasets, and 97.74% for the test dataset. The confusion matrices for the second healthy subject (HS02) demonstrated a similar result with the overall accuracy of 96.7% for the remaining data from the training dataset and average classification accuracy of 97.1% for the test dataset. For HS02 the most reliable class was the relaxed pose with an accuracy of 100% and extension pose had an average accuracy of 95%.

This result ensured us that the classification accuracy obtained from the unused training datasets could accurately estimate the classification performance in other conditions, and the ANN trained using three datasets enabled sufficiently accurate classification (>96%). Therefore, to prevent fatigue and decrease the overall duration of the experiment for the SCI subjects, we only collected three training datasets from SCI subjects.

#### SCI subjects

**Figure 9.**
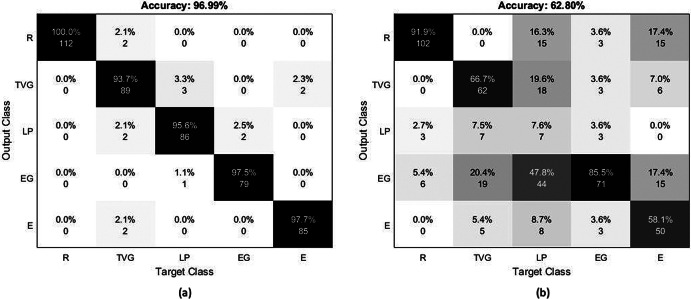
Confusion matrix for (a) S02 and (b) S04 during training sessions based on randomly selected unused training data. R, TVG, LP, EG, and E are relaxation, transverse volar grip, lateral pinch, extension grip, and extension, respectively.

Figure 9 presents the confusion matrices for the five target poses utilizing three sEMG sensors during the training sessions for S02 and S04. S02 showed the best classification results with an average accuracy of 96.99% (Figure 9a). The highest value was acquired for extension with 97.7% accuracy and the lowest value was obtained for the transverse volar grip with 93.7% accuracy (note that we did not consider relaxation for this comparison). Figure 9b shows the worst accuracy with an average accuracy of 62.80% for S04. The lateral pinch showed the lowest accuracy of 7.6%. The extension grip was the most successful pose (excluding relaxation) at 85.5% accuracy. The S01 and S03 had 88.8 and 88.2% average accuracy, respectively. The classification performance varied based on the conditions of the subjects.

### Probabilistic approach

**Figure 10.**
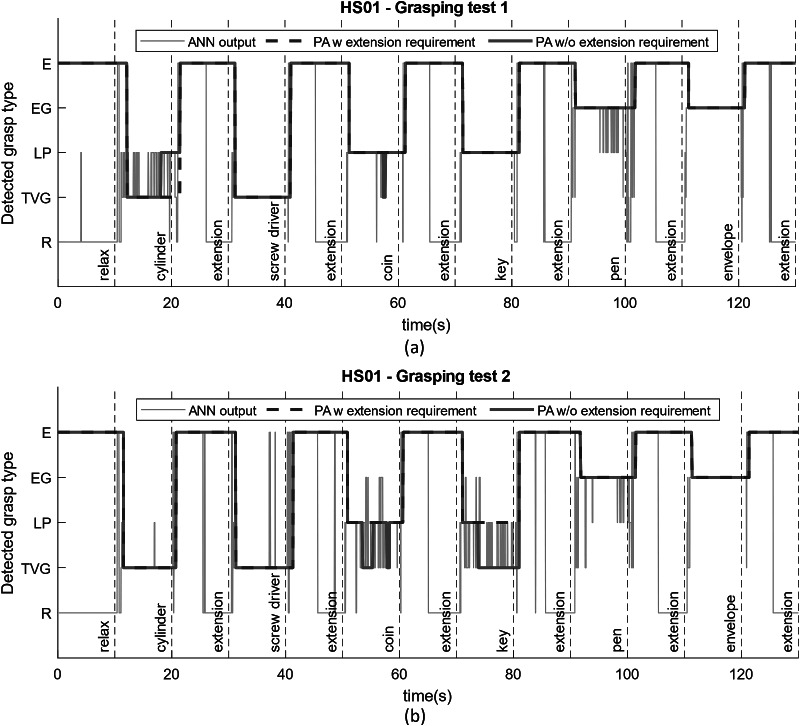
Reliability of the probabilistic approach for selecting the correct hand pose command for the robot shown in two different tests for a healthy subject (HS01). The light blue line is the ANN output without implementing the majority vote classifier or probabilistic approach. The pink line implements the probabilistic approach without requiring to switch to extension pose between two consecutive grasping poses, and the dark blue dashed line is our proposed probabilistic control approach for robust operation.

We tested the performance of the proposed probabilistic approach on healthy subjects during dynamic interaction with objects used in daily activities. Figure 10 shows the classification results for HS01 calculated based on three methods. The light blue line shows direct output of the ANN classifier without implementing majority vote classifier or the probabilistic approach. This output is noisy and unsuitable for selecting control commands for the robot. The pink line shows the output of the program for the detected grasp type while considering the majority vote classifier and the strategy to maintain the grasp after relaxation. However, it does not include the requirement to perform extension before switching to a different grasping pose. In this case, the threshold for the majority vote classifier was chosen to be 80%. In both tests, this strategy helped to reduce the noise compared to ANN output and detected the intended grasp type correctly in majority of cases. However, during the grasp of some objects (cylinder, coin, and key), the grasp type was misclassified by the ANN and the detected grasp was switched. This phenomenon is not desirable during real-time operation with the robot as the unwanted movement of the robot during manipulation can cause to drop the object and pose safety concerns for the user.

On the other hand, implementing the extension requirement as explained in the probabilistic approach along with the majority vote classifier helps to ensure robustness and safety through correct detection of grasp type. In Figure 10a,b, the dark blue dashed lines show the detected grasp type using the proposed probabilistic approach. In both tests, this method was able to successfully detect the appropriate intended grasp type and no misclassification occurred during grasping or extension. One drawback of using a majority vote classifier is the inherent delay it can cause before initiating a hand pose. However, comparing the delay values measured during the grasping test (Figure 10) with the delay values for initiating muscle activation during training of ANN (Figure 5), we can see that the large portion of it is due to the reaction time of the human to initiate movement after been shown a visual cue. The additional delay caused by the algorithm on average accounts for only addition of 0.22 s of delay compared to 1.08 s of average delay caused by human control (Figure 5).

**Figure 11.**
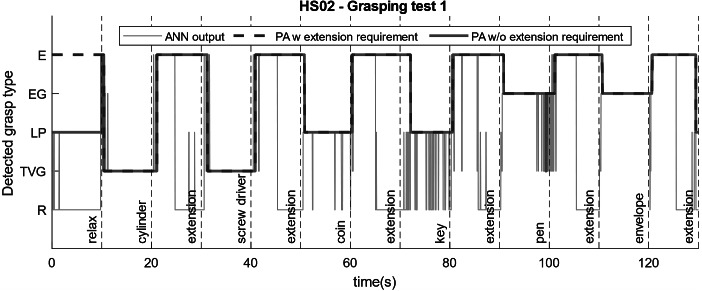
Reliability of the probabilistic approach for selecting the correct hand pose command for the robot shown in two different tests for the second healthy subject (HS02).

For HS02, one example of a grasping test is shown in Figure 11. We realized the proposed approach with a threshold of 60% could yield appropriate grasping commands for the second healthy subject, reducing the delays in the detection of the grasp and operation of the robot to 0.89 s since the visual cue. Similarly, the ANN output alone appears to be very noisy and misclassification occurs at many instances between relaxed pose and lateral pinch, and between transverse volar grip and extension grip. However, the probabilistic approach reduces the number of misclassifications to two instances during this experiment. And finally utilizing the probabilistic approach with the extension requirement detects the intention appropriately throughout the test and eliminates any instances of involuntary pose change.

### SHFT scores

The scores for the SHFT listed in Table 3 for SCI patients show that the hand functions of S01, S02, and S03 were improved by Maestro, but the hand function score of S04 was unchanged. For S01, the SHFT score without the exoskeleton was 41, first SHFT score with the exoskeleton was 47, and second SHFT score with the exoskeleton was 50. S01 had difficulty with active flexion of the fingers and abduction/adduction of the thumb. Maestro helped him to generate flexion of the fingers and abduction of thumb. He performed tasks with the correct hand grip and generated sufficient hand strength for ADL. For example, his scores were improved in lifting an iron, pouring water from a pure-pak carton, and writing with a pen. S01 had limited sensory feedback from his fingertips, which made it difficult to perform delicate tasks, especially without visual feedback, including picking up coins from a purse mounted on a wall and picking up nuts and putting them on bolts.

For S02, the SHFT score increased from 45 to 49 by using the exoskeleton. S02 had stiff-flexed fingers and difficulty with finger extension. He typically wrapped his hand around an object by pushing on his fingers and opened his hand by utilizing the other hand or contacting an object. When using Maestro, he could extend his fingers more easily and achieve higher scores for lifting an iron and pouring water from a jug, cup, and pure-pak carton. However, he achieved lower scores in tasks that required pinching small objects, such as unlocking a Yale lock with a key and writing with a pen.

For S03, the SHFT score increased from 40 to 41 by using the exoskeleton. S03 had a more severe SCI than S01 and S02, meaning the available EMG signals were weaker and more sparse than those of S01 and S02. The weaker EMG signals resulted in difficulty operating the exoskeleton with precise timing, leading to lower SHFT scores compared to S01 and S02. However, the exoskeleton did increase his grasping strength, allowing him to lift heavy objects, such as an iron and pure-pak carton containing water.

For S04, the SHFT score was the same with and without the exoskeleton. His injury was the most severe injury among the subjects who participated in our experiments. His EMG signals for the target task-related muscles were extremely weak, making it difficult to operate the exoskeleton. He was able to generate signals only for the extension grip and the control between target hand poses was very unstable. However, it was remarkable that S04 showed the most significant satisfaction among the subjects when achieving movement of his hand based on his intentions, even though the control was unstable.

## Discussion

We evaluated the hand function of four SCI patients in ADL with Maestro. Maestro, which is a light, comfortable, and compliant hand exoskeleton, was reliably controlled by the SCI patients. This study demonstrated the feasibility of an sEMG-driven hand exoskeleton in providing assistance for hand-grasping requirements in ADL. Hand function was improved overall with Maestro, but the results from the SHFT showed different values depending on the conditions of the SCI patients.

### EMG-driven Maestro control

The outcomes of this study can be used as guidelines for designing and controlling assistive devices for the SCI patients. We demonstrated that by taking advantage of compliance in the design of hand exoskeletons, the number of essential target hand poses can be reduced, resulting in easier and more robust control of the device in real-time.

Grasping skills mainly rely on independent abduction/adduction of the thumb and flexion/extension of the thumb and fingers (Tavakoli et al., 2015; Montagnani et al., 2016). Therefore, optimizing the number of target poses is a crucial component for an sEMG-driven hand orthosis. In this study, we utilized only three grips to perform SHFT (Sollerman and Ejeskär, 1995) and two additional poses (extension and relaxation) were utilized for Maestro control. This approach resolved the trade-off between complexity and performance of the system when designing the hand exoskeleton. However, the Multi-DoF nature of the robot helped with customizing the grasp parameters for each subject.

We aimed to use a minimal number of task-relevant muscles to make the use of the assistive robot simple and intuitive. Our C5–C7 level SCI subjects often had sufficient control on their muscle activity in these muscles to achieve acceptable classification accuracy. However, in case a subject is specifically weak in a certain muscle, the sensors can be moved more proximal to the body to target stronger muscles for robot control. It is important to note that electrode placements are important in robust sEMG-based intention recognition. Kim et al. proposed a simple compensation method for the rotation of electrodes and validated that only single motion can be used to compensate the electrodes shift (Kim et al., 2018).

To guarantee a stable operation, a majority vote classifier method has been used in many studies (Thielbar et al., 2017; Lu et al., 2019). Lu et al. (2019) showed that 12 SCI patients could control a hand exoskeleton using myoelectric pattern recognition for six motions. The results showed that control accuracy of an exoskeleton hand was higher than online sEMG classification accuracy without the device because of the majority vote technique used in the exoskeleton control. To reliably control a robotic hand, classification of a user-intention using sEMG signals of the SCI subject and majority vote technique for generation of input commands to an exoskeleton should be considered simultaneously.

In addition to the majority vote classifier, we implemented a number of imposed conditions on the proposed control algorithm to ensure additional robustness and safety. Apparent from the classification results in both healthy and SCI population, the extension hand pose was usually the most easily recognized by the classifier. Firstly, to prevent the risk of unwanted changes between two grasping poses (as a result of misclassification), we required the subjects and the control program to transition through extension pose before switching to a different grasping pose. This reduces the risk of unwanted movent during object interaction and dropping the object being manipulated. Secondly, we allowed remaining in a selected hand pose once the hand pose was selected through the majority vote classifier and the subject began to relax their muscles. This helped SCI subjects to stay relaxed after they have extended their fingers or after they have successfully grasped an object. In long-term, this policy helps to prevent fatigue, exhaustion, and misclassification due to fatigue.

To develop an assistive device that can be practically used by SCI patients, several technical problems need to be resolved. Although we have observed that the grasping performance of SCI subjects were improved with Maestro, the hand function scores did not improve dramatically. The main reason reported in the survey was the large size of Maestro. The compliance of the exoskeleton needs to be selected in a systematic way (Dollar and Howe, 2010). Selecting an adequate compliance which is optimal for the subject and completion of tasks may allow subjects to perform tasks more efficiently. Currently, soft robotic devices for improving hand function in SCI patients have been reported (Zhou et al., 2019; Tran et al., 2020). Soft exoskeletons are deformable to accommodate the shape of the hand with flexibility for operation. Furthermore, actively controlled variable stiffness may bring additional advantages.

### Effects of individual conditions of SCI patients

Significantly different muscle activation strategies with and without the exoskeleton might affect the classification accuracy. As the amount of time after injury increased, the improvement in SHFT scores was lower with the robot in this study, reflecting the habits and strategies SCI subjects have adopted for completing their daily grasping tasks in ADL. Based on the SHFT scores, two subjects (S01/S02) showed improvement when they utilized Maestro, whereas the other subjects (S03/S04) achieved similar results with and without Maestro. The degree to which a subject could independently move their body determined their performance on the SHFT. Among the four SCI patients, S01/S02 had the shortest times after injury and less paralysis of the wrist and fingers compared to the other subjects. Relatively long times for S03/S04 since their injuries can be one of the reasons why there was no change in the SHFT score. However, remarkable point was that two subjects showed comparable scores when they used their own grasping strategy without Maestro and used Maestro using sEMG despite of the short practice time for Maestro control. They could perform the grasping tasks using our EMG-driven methodology.

The scores of S03 with and without the exoskeleton were similar at 40 and 41. However, the exoskeleton increased his grasping strength, allowing him to lift heavy objects, such as an iron (3–4 SHFT scores) and pure-pak carton filled with water (2–4 SHFT scores), utilizing the correct grip (Table 3). For S04, who had the most severe injury among the subjects, he was able to reliably generate sEMG signals only for the extension grip and the control between the target hand poses was unstable because of weak EMG signals. However, S04 expressed the most satisfaction among the subjects when achieving movement of the hand based on his intentions, even though the control was unstable. Although two patients (S03/S04) showed no change in their SHFT scores, both indicated that they felt more comfortable with Maestro.

It is also important to note that the successful completion of some of the ADL tasks used in the SHFT requires not only the ability to move the fingers but also strength and mobility in upper arm and shoulders. For instance, lifting an iron or pouring water from a jug actively requires the involvement of upper arm and shoulders. For subjects with severer injuries, according to the collected footage of the experiment, although Maestro could provide enhanced grasping strength to hold iron and milk carton using the correct grip, the weakness in the upper arm and shoulders prevented successful completion of the task. Depending on the individual’s injury (especially when the injury affects higher parts of the spinal cord such as C4–C5) an assistive hand device alone might not be sufficient to fulfill all ADL needs without upper arm support.

### Comparison to state-of-the-art assistive systems

This study is the first in authors’ knowledge that has practically and systematically validated the use of an EMG-driven assistive hand device in ADL for spinal cord injury patients using a standardized hand function test. We demonstrated for the first time the feasibility of performing real-time control of assistive hand exoskeleton through SCI subject’s intention in a setting simulating the grasping and manipulation tasks in ADL. A large number of the previous assistive studies for the hand have either limited the hand function to only opening or closing, have not controlled the robot in an online manner using subject’s intention, or have limited the performance evaluation to few selected tasks that are not representative of daily activities.

In a recent study, soft robotic gloves have been proposed to help SCI patients (Cappello et al., 2018; Zhou et al., 2019; Tran et al., 2020). Zhou et al. (2019) provide a simple control strategy from the integrated soft sensors to detect relative changes in hand-object interactions for pinch flexion and power flexion. They showed that grasping force was improved for three patients. Tran et al. (2020) proposed a voice-controlled and tendon-actuated exoskeleton for four degrees-of-freedom of the hand. A case study with SCI patient was performed including the Box and Block Test and Jebsen-Taylor Hand Function Test. Cappello et al. (2018) utilized a fabric-based soft robotic glove with two operating modes for finger extension and flexion. For performance evaluation Toronto Rehabilitation Institute hand function test (TRI-HFT) was used, that includes grasping and releasing 11 daily objects, and measuring grasp strength. However, the control of the assistive glove was done by the researcher running the robot, and not based on the intention of the subjects.

Ngeo et al. (2013) have proposed a method for controlling joint angle of the index finger of subjects based on EMG activation of the opposite hand. However, they only validated their method with a healthy subject. In a study by Soekadar et al. (2016), authors have used EEG for detecting the intention of SCI subjects for hand movements, and an exoskeleton to help them with ADL. They only performed opening and closing function of the fingers and evaluated the performance of their method using the TRI test, which focuses on grasping and holding objects as opposed to performing functional tasks with daily objects. In an earlier study, Lucas et al. (2004) have used EMG to control pinching motion of a 1DoF device in SCI patients. Besides limitation of finger movement and grasping mode to only pinching, the performance evaluation was done through a few objects, and not for daily activities. Randazzo et al. (2018), propose an EEG-controlled hand exoskeleton for daily activities through power and precision grasps. However, they have not yet tested their device and control method on SCI subjects through a standard hand function test.

Our study has proven the feasibility and effectiveness of using EMG-driven multi-DoF assistive hand exoskeleton in daily activities. We believe the result of this study is a significant achievement for the assistive device community and hope to see many more examples of subject studies assessing the practicality of the assistive devices in real-time for use in daily activities.

### Study limitations

In this study, a grasping test was conducted to determine if subjects could hold objects with the correct grips and a researcher evaluated whether or not grasping was successful. Although the subjects with Maestro were able to perform grip tasks listed in Sollerman and Ejeskär (1995) utilizing the target hand poses, the success of grasping does not guarantee that subjects are able to perform the required tasks with the objects as instructed by SHFT. There is a difference between grasping an object and performing a task with that object.

The order of both conditions (with Maestro and without Maestro) were the same for all subjects: (a) with Maestro and (b) without Maestro (bare hand). The reason the bare hand condition (without Maestro) was performed second was to minimize the learning effect on SHFT. Different grasping strategies were used for the two conditions. For the first condition with Maestro, selected target poses were used to perform 20 tasks of SHFT. For the second condition without Maestro, their own strategy was used depending on individual because some of patients could not perform the task using the selected poses. Therefore, the order used in this study did not increase the SHFT scores for Maestro control, but it could positively affect the SHFT scores for the bare hand condition due to learning effect. Even without pre-knowledge about SHFT, we expected that the condition with Maestro showed better performance based on SHFT scores.

Only short-term training was utilized for most subjects in this study. SHFT scores in this study could be varied depending on the patient compliance with increasing practice time because the EMG classification can be improved by user learning (Bunderson and Kuiken, 2012). Improvement through repetitive training indicates that the effects of learning and experience with the exoskeleton system are important. S01 showed the most noticeable improvement in terms of SHFT results when he completed two consecutive tests, as shown in Table 3 suggesting learning effects on the exoskeleton control.

For quantitative evaluation of the hand function, we used the SHFT scores based on the time spent for completing tasks, number of drops, and hand pose while performing daily activities with objects. However, SHFT results are not able to reflect possible therapeutic effects on the hand function such as improved hand function of bare hand, physiological biomechanics, and inhibition of compensatory movements. Due to short-term training and experiments, this study could provide a feasibility of EMG-driven hand assistive device. As Thielbar et al. (2017) showed the therapeutic benefits of EMG-driven hand device in a longitudinal intervention, proposed method needs to evaluate for more subjects with multiple training sessions.

**Figure 12.**
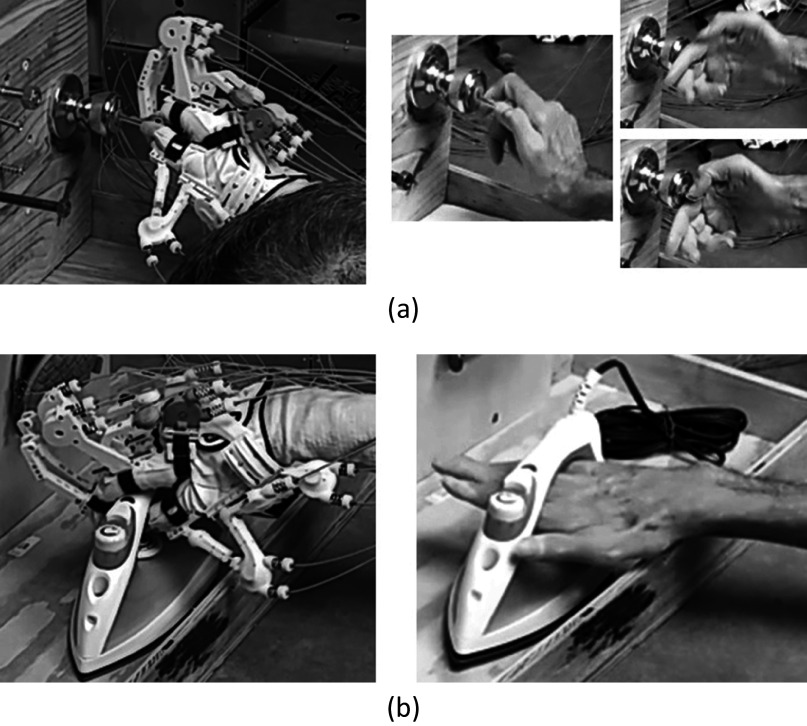
Hand poses with Maestro (left) and without Maestro (right) during (a) pick up key, put into Yale-lock and turn 90° and (b) lift iron over edge for S01.

Although the SHFT score helped to analyze the hand function with a quantitative index, monitoring changes in arm and body motion can be used to analyze how compensatory movements are used during grasping tasks with and without the assistance of Maestro. For example, Figure 12 showed how a subject performed the same task differently under two test conditions. During the task of “pick up key, put into Yale-lock, and turn 90°,” S01 used the appropriate pinch pose with Maestro, but had to switch several compensatory hand poses to perform this task without Maestro (Figure 12a). The SHFT scores were 1 for both conditions, which did not necessarily reflect this difference. During the task “Lift iron over edge,” S01 used the pinch poses with Maestro and a compensated mixture of hand poses without Maestro (Figure 12b). However, the SHFT score without Maestro (4) was greater than that of the condition with Maestro (2). Montagnati et al. used motion analysis test to assess the movements of the trunk, right shoulder, and arm during grasping tasks (Montagnani et al., 2015, 2016). The compensatory movements of body segment angles were used as the index for evaluating the performance. Variations in the appropriate kinematics of motions combined with the SHFT score could provide additional qualitative information regarding the hand function.

The selection of sEMG sensors can be improved. Because the selection did not consider the symptoms of individual SCI patients, the sensor locations were modified for each individual SCI. Every SCI patient has different symptoms and thus some patients have particularly available muscle signals. Considering the individual perspective of SCI to select the locations of sEMG sensors in an iterative manner will lead to improved results.

## Conclusion

The main goal of this study was to evaluate the feasibility of the developed hand exoskeleton called Maestro for performing the hand function of SCI patients using the proposed control method. An EMG-based control system was implemented to classify four target poses and relaxation utilizing three sEMG sensors on the forearm and palm. We evaluated the accuracy and robustness of the proposed control method taking advantage of a probabilistic approach in classifying the intention and selecting the appropriate robot command in healthy subjects during real-time dynamic grasping. Four SCI patients performed a standardized hand function test, called SHFT, and their grasping performance improved with Maestro on the SHFT scores. The results confirm the potential of Maestro to improve the hand function of SCI patients and the subjects indicated significant satisfaction in achieving tasks based on their intentions. By demonstrating reliable control of multiple joints with signal from numerous muscles, we have advanced the field of EMG-driven manipulation control for SCI patients. Controlled grasping for daily objects also paves the way for a more practical and applicable assistive device that will positively affect the day-to-day life of SCI patients.
